# Mitochondrial Medicine in the COVID-19 Era

**DOI:** 10.3390/jcm10225235

**Published:** 2021-11-10

**Authors:** Daniele Orsucci

**Affiliations:** Unit of Neurology, San Luca Hospital, Via Lippi-Francesconi, 55100 Lucca, Italy; orsuccid@gmail.com or daniele.orsucci@uslnordovest.toscana.it

**Keywords:** CPEO, Leber, Leigh syndrome, MELAS, MERRF, mitochondrial myopathy, MNGIE, mtDNA, NARP, PEO

Mitochondrial disorders are a remarkably complex group of diseases caused by impairment of the mitochondrial respiratory chain (or electron transport chain). Phenotypes may range from isolated myopathy to multisystem disorders, with highly variable age of onset, severity, and progression. The genetic defect can be located in the mitochondrial (mtDNA) or the nuclear genome (nDNA). Mitochondrial diseases due to mtDNA mutations are peculiar in human genetics, following different inheritance laws than nuclear mutations [1].

If we consider that muscular involvement is the core feature of these disorders and that transient muscular dysfunction is common during COVID-19 even in the general population [2], we can easily understand the increased concerns of mitochondrial patients during the pandemic period.

However, by searching PubMed with the terms “mitochondrial, COVID* OR mitochondrial, coronavirus*”, to date (3 November 2021), there is no empirical evidence of a heightened risk for a severe outcome of COVID-19 in these subjects, except from an isolated report of a patient with pre-existing severe MELAS (mitochondrial encephalomyopathy, lactic acidosis, and stroke-like episodes) further complicated by COVID-19 [3].

A retrospective Italian case series on 27 mitochondrial patients infected by SARS-CoV-2 revealed that most patients had mild infections (85%) and were treated at home. Only one patient died because of COVID-19 pneumonia. Therefore, individuals with a mitochondrial disorder appeared to be similarly affected by SARS-CoV-2 infection compared with the general population [4].

The COVID-19 pandemic may also have had indirect effects on mitochondrial patients, e.g., by losing the multisystem follow-up checks or even by increasing alcohol consumption and the number of cigarettes smoked during the recent lockdowns. This latter factor may have led to the clinical expression of a previously asymptomatic Leber hereditary optic neuropathy in at least some individuals [5].

Patient care standards for mitochondrial disorders, including, for instance, cardio-respiratory and nutritional follow-up, must also be provided during pandemics (with telemedicine facilities when appropriate). Furthermore, mitochondrial patients must be offered age-appropriate vaccination [6], including vaccines against COVID-19, that are as safe for them as for the general population.

In this context, this Special Issue (“Mitochondrial Disorders: Molecular, Clinical and Therapeutic Advances”, https://www.mdpi.com/journal/jcm/special_issues/Mitochondrial_Clinical) (accessed on 1 November 2021). aims to clarify the molecular basis, the clinical spectrum, the diagnostic approach, and the treatment advances of mitochondrial disorders. We do not fully know mitochondrial diseases yet, but we are beginning to unravel their complexity.
